# Nurses’ knowledge and attitude towards children pain management: a multi-site survey study

**DOI:** 10.3389/fped.2023.1182529

**Published:** 2023-06-23

**Authors:** Efrem Fenta, Simegnew Kibret, Metages Hunie, Tadese Tamire, Denberu Eshetie, Shimelis Seid, Yewlsew Fentie, Eleni Amaha, Tiruwork Desse, Bantayehu Dejen, Keder Essa, Diriba Teshome

**Affiliations:** ^1^ Department of Anesthesia, College of Health Sciences, Debre Tabor University, Debre Tabor, Ethiopia; ^2^ Department of Anesthesia, College of Health Sciences, Addis Ababa University, Addis Ababa, Ethiopia; ^3^ Department of Internal Medicine, College of Health Sciences, Debre Tabor University, Debre Tabor, Ethiopia; ^4^ Department of Nursing, Debre Tabor Health Sciences College, Debre Tabor, Ethiopia

**Keywords:** nurses, knowledge, attitude, children, pain management

## Abstract

**Background:**

Adequate children’s pain management is universally considered an ethical obligation. In evaluating and treating children’s pain, nurses invest more time and take a leading role. This study aims to evaluate the knowledge and attitudes of nurses towards the treatment of pediatric pain.

**Materials and Methods:**

A total of 292 nurses working at four South Gondar Zone hospitals of Ethiopia was surveyed. To gather information from study participants, the Pediatric Nurses’ -Knowledge and Attitudes- Survey Regarding Pain (PNKAS) was employed. Frequency, percentage, mean, and standard deviation of the data were used for descriptive analysis, while Pearson correlation, one-way between-groups analysis of variance, and independent-samples t-test were used for inferential analysis.

**Results:**

A large percentage of nurses (74.7%) lacked adequate knowledge and attitudes (PNKAS score <50%) for pediatric pain treatment. The mean ± SD accurate response score of 43.1% ± 8.6% was achieved by nurses. An increase in pediatrics nursing experience was significantly correlated with nurses’ PNKAS score (*p* < 0.001). The mean PNKAS scores of nurses who had official pain management training differed in a statistically significant way as compared to its counterpart (*p* < 0.001).

**Conclusion:**

Nurses who are working South Gondar Zone of Ethiopia have insufficient knowledge and attitudes towards treatment of pediatric pain. Therefore, pediatric pain treatment in-service training is urgently needed.

## Introduction

Pain has been defined as an unpleasant sensory and emotional experience resulting from actual or potential tissue damage (1). A fifth vital sign, pain, was added by the American Pain Society (2, 3).

Children range from preterm newborns to teenagers. Age-related changes are clearly visible that affect pain assessment and management due to differences in physiology and pharmacology with adults (4).

Children’s pain could be assessed based on the child’s age and communication ability, using self-report, behavioral observation, or physiological data. The standard method of assessing pain is through self-report due to the subjective nature of pain, rather than focusing on the patients’ behavioral observation; however, it is difficult for neonates, infants, and young adolescents (5, 6). Individual self-report is often favored especially in children between the ages of 3 and 7 who are able to give information on the location, quality, and intensity of the pain (7, 8). Pain scales available for newborns, babies, kids, teenagers, people with speech impairments, and others. These include the Wong-Baker Faces Pain Rating Scale (9), Visual analog scale (VAS) (9), Numeric Rating Scale (NRS) (10), Pediatric Pain Questionnaire (11), and Colorado Behavioral Numerical Pain Scale (for sedated patients) (12).

According to estimates, 35%–85% of people experience pain (5). According to reports, 50% of patients suffer moderate pain, while 30% of patients report severe pain. Up to 40% of patients have inadequate pain management in daily practice (13).

All patients have a right to optimal pain management, and all medical practitioners have a duty to provide it. Most of the time, nurses are in charge of evaluating and treating children’s pain, who also provide care that is adequate for the patient’s needs (9, 14, 15). Overall patient outcomes are improved when pediatric pain treatment is approached with a sufficient knowledge and a positive attitude (2, 16, 17). It’s unacceptable and unethical to fail to maintain proper pain control. Healthcare practitioners’ lack of understanding of the evaluation and treatment of pain leads to ineffective pain therapy (18, 19). The challenges to pain management by nurses worldwide are negative attitudes, inadequate recordkeeping, inadequate patient assessment, and improper analgesic use (20, 21). From our observation, the practice of children’s pain management is inadequate and there is no evidence regarding knowledge and attitude towards children’s pain management in Ethiopia. Therefore, the purpose of the study is to evaluate nurses’ knowledge and attitudes on the treatment of pediatric pain in South Gondar Zone Hospitals of Ethiopia.

## Materials and methods

### Design and setting

This cross-sectional study was carried out in four South Gondar Zone Hospitals (Addis Zemen Primary Hospital, Nefas Mewucha Primary Hospital, Dr. Ambachew Mekonnen Memorial Hospital, and Debre Tabor Referral Hospital) from 1 to 30 January, 2021.

### Participants

There were about 335 nurses (Addis Zemen had 45, Nefas Mewucha had 74, Dr. Ambachew Mekonnen Memorial had 27, and Debre Tabor Referral Hospital had 189), according to a report from the South Gondar Zone Health Bureau. We surveyed every nurse available during the data collection period. This study included nurses who worked at South Gondar Zone Hospitals during the data collection period; involuntary nurses, students, and nurses with less than six months of experience on the job were excluded.

### Operational definitions

**Child:** An individual less than 18 years of age (4).
**Sufficient knowledge and attitude:** When a nurse’s PNKAS score for managing children’s pain was 50% or above (22).
**Insufficient knowledge and attitude:** When a nurse’s PNKAS score for managing children’s pain was less than 50% (22).

### Instruments

Sociodemographic form: The questionnaire consists of 12 questions related to socio-demographic and job-related questions, like age, sex, education level, and nursing experience.
**Pediatric Nurses’ Knowledge and Attitudes Survey Regarding Pain:** The questionnaire consists of 42 questions (25 true/false, 13 multiple choices, and 2 case studies) about knowledge and attitudes regarding pediatric pain management (PNKAS regarding pediatric pain tool).

The PNKAS tool, which was designed in 1987 and has been frequently used since then and been refined over time, was employed. The review by the pain experts has proven the validity and reliability of the content. The American Pain Society, the World Health Organization, and the National Comprehensive Cancer Network Pain Guidelines all contributed to the tool’s content. Avoiding labeling items as evaluating knowledge or attitudes will be most useful when analyzing the data. There are many items that evaluate both knowledge and attitudes. The highest possible raw score on the PNKAS tool was 42, which corresponds to a 100% accurate response. Each question received a score of “1” when it was answered correctly and “0” when it wasn't. The mean score and total percentage score were calculated by analyzing and formulating the scores (23).

### Data collection

Data was collected from available nurses during the data collection period by trained personnel who administered the questionnaire after obtaining a permission letter from South Gondar Zone Hospital administrative bodies. Self-reporting was utilized for collecting the data, and participants in the study were asked to fill out the form within 10 min.

### Data analysis

After being coded and being verified as complete, the data were entered into SPSS version 23. The descriptive analysis of the data employed frequency, percentage, mean, and standard deviation, while the inferential analysis used Pearson correlation, one-way between-groups analysis of variance, and independent-samples *t*-test. Statistical significance was considered as a *p*-value less than 0.05.

## Results

### Demographic characteristics of the nurses

This survey was completed by 292 nurses in total, with an 87% response rate. The nurses’ ages ranged from 22 to 50 years, with a mean age of 31 years and a standard deviation of 6 years. More nurses (53.4%) were enrolled in the Bachelor’s degree program. More nurses (*n* = 222, 76.0%) had up to five years of experience in the field, compared to fewer nurses (*n* = 70, 24.0%), who had more experience. The majority of the nurses (82.5%) did not have official pain management training, and 72.6% didn’t take pain education as part of the course syllabus (Table 1).

**Table 1:** Demographic and job-related characteristics of the nurses (*N* = 292).

Characteristic	Frequency	Percentage
**Sex**		
Male	141	48.3
Female	151	51.7
**Age in years**		
20–29	156	53.4
30–39	102	35.0
≥40	34	11.6
**Educational level**		
Diploma	66	22.6
Bachelor’s degree	156	53.4
Master’s degree	70	24.0
**Monthly income in Ethiopian birr**		
3,000–6,000	58	19.9
6,000–9,000	146	50.0
9,000–12,000	88	30.1
**Years of nursing experience**		
0–5	222	76.0
>5	70	24.0
**Years of pediatrics nursing experience**		
0–5	223	76.4
Greater than 5	69	23.6
**Have you an official pain management training**		
Yes	51	17.5
No	241	82.5
**Did you take a course that included pain education?**		
Yes	80	27.4
No	212	72.6
**Have you specific protocols about pediatrics pain management in your institution**		
Yes	124	42.5
No	168	57.5
**Have you read any guidelines for management of pediatrics pain**		
Yes	97	33.2
No	195	66.8
**Your current working area**		
Outpatient department	76	26.0
Emergency department	57	19.5
Pediatrics ward	45	15.4
Medical ward	77	26.4
Surgical ward	37	12.7
**Work overload (average working hours per day)**		
Less than 8 h	129	44.2
9–16 h	86	29.5
Greater than 16 h	77	26.4

### Nurses’ knowledge and attitudes regarding pediatrics pain management

Nurses scored an average of 18.1 ± 3.6 out of 42, or 43.1% ± 8.6% out of 100%, on the 42-item Knowledge and Attitude component of the PNKAS tool, ranging from a minimum score of 12 (28.6%) to a maximum score of 26 (61.9%). A greater proportion of the nurses (*n* = 218, 74.7%) had insufficient knowledge and attitude towards pediatric pain management. The most frequently correctly answered question was “After an initial dose of an opioid analgesic is given, subsequent doses should be adjusted in accordance with the individual patient’s response”, followed by “Presence of parents isn’t necessary during painful procedures” (Table 2). The most frequently incorrectly answered question was “Analgesics for continuous pain should be given” followed by “The recommended route of administration of opioid analgesics to children with brief, severe pain of sudden onset” (Table 3).

**Table 2:** Top 5 items most often answered correctly by nurses (*n* = 292).

Item content (correct answer)	Frequency (%) correct
After an initial dose of opioid analgesic is given, subsequent doses should be adjusted in accordance with the individual patient’s response (T).	175 (59.9)
Presence of parents isn’t necessary during painful procedures (F).	164 (56.2)
Non-pharmacological pain management is effective in mild and moderate pain while rarely useful in severe pain (F).	163 (55.8)
If the infant/child/adolescent can be distracted from his pain, this usually means that he is not experiencing a high level of pain (F).	162 (55.5)
If the source of the patient’s pain is unknown, opioids should not be used during the pain evaluation period, as this could mask the ability to correctly diagnose the cause of pain (F).	159 (54.5)

**Table 3:** Top 5 items most often answered incorrectly by nurses (*n* = 292).

Item content (correct answer)	Frequency (%) incorrect
Analgesics for continuous pain should be given (around the clock on a fixed schedule)	240 (82.2)
The recommended route of administration of opioid analgesics to children with brief, severe pain of sudden onset (intravenous)	239 (81.8)
A child with chronic cancer pain has been receiving daily opioid analgesics for two months. The doses increased during this time period. Yesterday the child was receiving morphine 5 mg/hour intravenously. Today he has been receiving 10 mg/hour intravenously for 3 h. The likelihood of the child developing clinically significant respiratory depression is (1–10%)	219 (75.0)
Your above assessment is made two hours after he received morphine 2 mg IV. After he received the morphine, his pain ratings every half hour ranged from 6 to 8 and he had no clinically significant respiratory depression, sedation, or other untoward side effects. He has identified 2 as an acceptable level of pain relief. His physician’s order for analgesia is “morphine IV 1–3 mg q1h PRN pain relief.” Check the action you will take at this time (Administer morphine 3 mg IV now).	215 (73.6)
Which of the following IV doses of morphine administered over a 4 h period would be equivalent to 30 mg of oral morphine given q 4 h?(Morphine 10 mg IV)	211 (72.3)

### Relation between nurses’ characteristics and their total PNKAS score

The mean PNKAS scores of nurses with less than 5 years of total nursing experience (M = 18.2, SD = 3.6) and nurses with more than or equal to 5 years of total nursing experience (M = 20.0, SD = 3.0) differed statistically significantly (*p* = 0.008) according to the results of an independent-samples *T*-test analyses. Also, there was a statistically significant difference in the mean PNKAS scores between nurses with less than 5 years of experience in pediatric nursing (M = 17.6, SD = 3.5) and nurses with more than or equal to 5 years of experience (M = 19.7, SD = 3.4), with a *p*-value of less than 0.001. The PNKAS scores of nurses and years of pediatric nursing experience were statistically significantly correlated (*p* 0.001). The difference between the mean PNKAS scores of nurses with official pain management training (M = 21.5, SD = 3.3) and nurses without official pain management training (M = 17.4, SD = 3.2) was statistically significant (*p* < 0.001). The other demographic and job-related characteristics of nurses did not differ statistically significantly from one another.

## Discussion

Pain management in children is a major challenge for all health care professionals due to inadequate knowledge and attitudes of pain assessment, treatment, and fears of adverse effects of analgesics, including respiratory depression (24).

According to this survey, nurses achieved a mean correct answer score of 18.1 out of 42, or 43.1% out of 100%, with a minimum correct answer score of 12 (28.6%) and a maximum correct answer score of 26 (61.9%). More nurses (74.7%) reported inadequate knowledge and attitudes (PNKAS score below 50%) regarding pediatric pain management. Similarly, a study conducted by Palermo et al. (24) in five governmental Hospitals in the Riyadh region of the Kingdom of Saudi Arabia with a total of 410 nurses revealed that poor overall knowledge and attitudes regarding pediatric pain management. The mean score achieved by nurses was 18.1 out of 40 or 45.2% out of 100%. Other studies conducted by *Ahmad Bajjali* (25), *Ayfer Ekim* (26), *Omar Al Omari* (27), *Amponsah et al*. (28) showed that nurses had insufficient knowledge and attitudes regarding pediatrics pain management. In contrary to our finding, a study conducted by *Ho SE* et al. (16) found the majority of the respondents (78.5%) were having a positive attitude towards pain management. According to a study by Tagele et al. (29), the majority of nurses had good knowledge and positive attitudes of pediatric pain management. These variations may be due to the fact that the studies were carried out in referral hospitals rather than primary hospitals, which might have lacked the resources and knowledge necessary for pediatric pain management.

This study revealed that an increased pediatrics nursing experience was significantly correlated with nurses’ PNKAS score (*p* < 0.001). There was a statistically significant difference in the mean PNKAS scores of nurses who had official pain management training as compared to its counterpart (*p* < 0.001). There were no statistically significant differences between all other items of nurses’ demographic and job-related characteristics (age, gender, education level, income, presence of pediatrics pain management protocols, and work overload) with their total PNKAS score. Similarly, a study conducted by *Ahmad Bajjali* (25), *Omar Al Omari* (27), *Amponsah* et al. (28) showed that demographic and job-related characteristics (gender, age, education level, presence of pain management protocols, and committees) had no significant relationship with nurses’ knowledge and attitude level. In line with our finding, *Palermo* et al. (24) revealed that there was a statistically significant correlation was found between years of pediatric nursing experience and PNKAS score (*p* = .009). Unlike our finding, *Ayfer Ekim* (26) reported that an increased work experience was not significantly correlated with the total PNKAS scores (*p* > .05).

The Pediatric Nurses’ Knowledge and Attitudes Survey Regarding Pain (PNKAS), which has been regularly updated, valid, and reliable as proved by the review of pain experts, may be the study’s strongest point. Its generalizability, reliability, and versatility may be additional virtue given that it is a survey study. We made an effort to keep recall periods to a minimum and employed a validated instrument. However, it is difficult to prevent recall and social biases, which could be seen as a limitation of this research. We recommend nurse educators to take into account pediatrics pain management courses when designing professional development courses, as well as for researchers looking to learn more about the barriers to providing pediatrics patient care.

## Conclusion

The results revealed that Ethiopia’s South Gondar Zone nurses’ knowledge and attitudes of pediatric pain management was insufficient. As a result, we recommend pediatrics pain management courses to take into account when designing curriculum programs for professional development courses, and ongoing in-service trainings to enhance nurses’ knowledge and attitude towards pediatric pain management.

## Data Availability

The original contributions presented in the study are included in the article/Supplementary Material, further inquiries can be directed to the corresponding author.
